# Therapeutic Efficiency of Nasal Mucosa-Derived Ectodermal Mesenchymal Stem Cells in Rats with Acute Hepatic Failure

**DOI:** 10.1155/2023/6890299

**Published:** 2023-01-09

**Authors:** Wen Xiang, Xinhe Wang, Xinghui Yu, Yan Xie, Li Zhang, Naiyan Lu, Wentao Jiang

**Affiliations:** ^1^School of Medicine, Nankai University, Tianjin, China; ^2^Department of Liver Transplantation, Tianjin First Central Hospital, Tianjin, China; ^3^School of Food Science and Technology, Jiangnan University, Wuxi, China

## Abstract

**Background:**

Liver transplantation is limited by the insufficiency of liver organ donors when treating end-stage liver disease or acute liver failure (ALF). Ectodermal mesenchymal stem cells (EMSCs) derived from nasal mucosa have emerged as an alternative cell-based therapy. However, the role of EMSCs in acute liver failure remains unclear.

**Methods:**

EMSCs were obtained from the nasal mucosa tissue of rats. First, EMSCs were seeded on the gelatin-chitosan scaffolds, and the biocompatibility was evaluated. Next, the protective effects of EMSCs were investigated in carbon tetrachloride- (CCl_4_-) induced ALF rats. Finally, we applied an indirect coculture system to analyze the paracrine effects of EMSCs on damaged hepatocytes. A three-step nontransgenic technique was performed to transform EMSCs into hepatocyte-like cells (HLCs) in vitro.

**Results:**

EMSCs exhibited a similar phenotype to other mesenchymal stem cells along with self-renewal and multilineage differentiation capabilities. EMSC-seeded gelatin-chitosan scaffolds can increase survival rates and ameliorate liver function and pathology of ALF rat models. Moreover, transplanted EMSCs can secrete paracrine factors to promote hepatocyte regeneration, targeted migration, and transdifferentiate into HLCs in response to the liver's microenvironment, which will then repair or replace the damaged hepatocytes. Similar to mature hepatocytes, HLCs generated from EMSCs possess functions of expressing specific hepatic markers, storing glycogen, and producing urea.

**Conclusions:**

These results confirmed the feasibility of EMSCs in acute hepatic failure treatment. To our knowledge, this is the first time that EMSCs are used in the therapy of liver diseases. EMSCs are expected to be a novel and promising cell source in liver tissue engineering.

## 1. Introduction

Acute liver failure (ALF) is a potentially fatal clinical syndrome caused by viral hepatitis, hepatotoxic drugs, immunoinflammatory etiology, and other factors, resulting in acute massive necrosis of hepatocytes and restricted regeneration [1]. Various medications and blood purification methods have been explored to aid in liver failure recovery, but the outcomes were usually poor. While liver transplantation is the most successful treatment for ALF, there are several clinical application restrictions, including donor unavailability, immunoreactions, and high healthcare costs [2].

In recent years, mesenchymal stem cells (MSCs), owing to their self-renewal and multiple differentiation potential including hepatic differentiation, have gained attention as a potential therapeutic approach in the treatment of liver failure [3]. MSCs' therapeutic effects are based mainly on their pleiotropic properties of paracrine-mediated tissue healing and their differentiation ability to repair damaged tissue and cells [4]. MSCs have been effectively extracted from a variety of organs. Typically, the bone marrow, adipose tissue, and umbilical cord, which could be induced to differentiate into hepatocyte-like cells (HLCs) [5], are regarded as the primary sources of mesenchymal stem cells for hepatic repair and regeneration. Also, many studies have shown that MSCs can improve hepatic function, downregulate hepatocyte apoptosis, and promote liver regeneration in animal models of ALF [6–8].

The lamina propria of the nasal mucosa lining the respiratory region contains neural crest-derived ectodermal mesenchymal stem cells (EMSCs) [9], which can be cultured and amplified in vitro while preserving their stem cell characteristics and multidirectional differentiation potential. Autologous transplantation of EMSCs offers some advantages [10] including abundant resources, convenient availability, and noninvasive damage while presenting no ethical concerns. Our prior studies indicated that EMSCs could differentiate into neural cells [11] and osteocytes [12] under specific induction conditions. The curative effect has been proved in rat models in spinal cord injury [13], nerve repair [11], and bone defect restoration [12]. However, the hepatic differentiation potential of EMSCs and their detailed contribution to liver failure remain unknown. Although MSC-based therapy has a high therapeutic potential for ALF, the number of functional and accessible MSCs after transplantation is restricted. The poor microenvironment in the injured tissue, including microcirculatory disturbance, inflammatory stimulus, poor oxygen, and nutrient supply limitation, makes it very difficult for exogenous MSCs to recruit and survive after transplantation [14]. To address these issues, many researchers attempted to deliver MSCs together with biomaterial scaffolds to aid in cell survival or tissue regeneration. Xu et al. reported that MSCs derived from fat or bone marrow preseeded on a silk fibroin scaffold could effectively repair acute liver failure in rats [15]. Chitosan is a kind of polycationic polysaccharide that originated from the marine. It was selected as an effective cell-loaded agent due to its favorable properties, such as nontoxicity, good biocompatibility, biodegradability, and excellent membrane forming ability [16]. In this study, EMSCs were used as seed cells and presented good biocompatibility with gelatin-chitosan (GC) matrices. Posttransplantation of EMSCs could remarkably restore liver injury in ALF rat models induced by carbon tetrachloride. We first successfully induced EMSCs to differentiate into HLCs in vitro with selective cell factors. These results show that EMSCs are expected to be an excellent source for the hepatocyte seed bank and function significantly in autologous stem cell transplantation in treating liver disease.

## 2. Materials and Methods

### 2.1. Animals

Sprague-Dawley (SD) rats (weight: 200-220 g, male) were provided by the Experimental Animal Center of Jiangnan University. All animal experimental protocols followed the guidelines of the National Institutes of Health (NIH) and were approved by the Institutional Animal Care and Use Committee (approval number: JN.No20220430S0800615). Climate-controlled conditions were provided with alternate 12 hours of light and dark cycles, as well as free access to regular food and water for the housed animals.

### 2.2. Preparation of Gelatin-Chitosan (GC) Matrices

GC matrices were prepared following protocol. Briefly, chitosan was dissolved with 2% acetic acid to achieve a final concentration of 1%. Then, 200 *μ*L of 1% genipin was slowly added into 5 mL chitosan solution and 5 mL 1% gelatin solution. The prepared composite was spread over a glass plate and subsequently dried overnight in a vacuum drying oven. The obtained GC matrices were fragmented into small pieces (2 × 2 cm^2^). Prior to application, the matrices were disinfected with 75% ethanol for 2 hours, rinsed three times in PBS, and treated in 1 mL of DMEM/F12 medium at 37°C overnight.

### 2.3. Isolation, Culture, and Expansion of EMSCs

The rats were deeply anesthetized by an intraperitoneal injection of phenobarbital sodium (200 mg/kg). The isolation of EMSCs was constructed based on the protocol described in our previous study [17]. Briefly, respiratory mucosa tissue samples from the lower two-thirds of the nasal septum were carefully isolated and completely sheared to pieces. The tissue fragments were incubated with DMEM/F12 medium containing 10% FBS, 2 mM L-glutamine, and 100 U/mL penicillin-streptomycin in plastic culture flasks at 37°C and 5% CO_2_. Cell morphological changes were observed every day under the inverted microscope, with the culture medium replaced every 3 days. The primary EMSCs were passaged at 80-90% cell confluence, and the cells in the third passage (P3) were used in all of our assays. Immunofluorescence staining was conducted to detect the expression of various stem cell markers, including vimentin and nestin.

### 2.4. Osteogenic Differentiation of EMSCs

The P3 EMSCs obtained from the logarithmic growth phase were inoculated on a 24-well plate at the density of 1 × 10^4^ cells/well. Osteogenic induction solution was added when cell fusion amounted to 80%. The culture medium was changed every 3 days, and alizarin red staining was performed 21 days later. The cell suspensions were fixed with 4% paraformaldehyde for 30 min, washed twice with phosphate buffer, and stained with alizarin red for 30 min. Then, the unbound dyes were washed off with distilled water. The stained bone nodules were observed with phase-contrast microscopy.

### 2.5. Adipogenic Differentiation of EMSCs

The P3 EMSCs were seeded at a density of 1 × 10^4^ cells/well in a 24-well plate and cultured with adipogenic induction medium for 21 days. Fat droplets of verified size within adipocytes differentiated from EMSCs could be observed with the Oil Red O staining method. Briefly, the cells were fixed with 4% paraformaldehyde for 15 minutes, washed three times with phosphate buffer and 60% isopropanol, and stained with Oil Red O solution for 30 min at room temperature. The staining cells were investigated under phase-contrast microscopy.

### 2.6. Hepatic Differentiation of EMSCs In Vitro

The third passage cultured cells were harvested at a density of 1 × 10^5^ cells/well until 70%-80% confluence was reached and then seeded in DMEM/F12 medium supplemented with 20 ng/mL epidermal growth factor (EGF, Sino Biological, China) and 10 ng/mL basic fibroblast growth factor (bFGF, Sino Biological, China) for two days. To induce hepatic differentiation, this study drew upon the previously reported three-step protocol designed for human umbilical cord-derived MSCs [18]. The step-1 induction medium consists of DMEM/F12 supplemented with 20 ng/mL hepatocyte growth factor (HGF, Sino Biological), 10 ng/mL bFGF (Sino Biological, China), and 0.61 g/L nicotinamide (NTA, Sigma-Aldrich, USA) for 7 days. Thereafter, the cells were treated by a maturation medium including DMEM/F12 supplemented with 20 ng/mL oncostatin M (OSM, Sino Biological, China), 1 *μ*mol/L dexamethasone (Dexa, Solarbio, China), and insulin-transferrin-selenium premix (ITS, Sigma-Aldrich, USA) for an additional week. The culture medium was replaced with fresh medium twice a week.

### 2.7. Live/Dead Staining

To determine biocompatibility between EMSCs and GC matrices in vitro, the live-dead cell staining kit (Biyuntian, China) was performed according to reagent instructions. Briefly, the 3rd passage EMSCs were seeded at a density of 1 × 10^5^ cells/cm^2^. Propidium iodide (1000x) and calcium-AM (1000x) were diluted to final concentrations of 500x, respectively. When the cell adherent growth and fusion reached 70%-80%, 300 *μ*L of the mixed solution was added to each well and stained at 37°C for 30 min. The distribution of cells and their viability were assessed using an inverted fluorescence microscope (Axio Vert A1).

### 2.8. Cell Counting Kit 8 (CCK8) Assay

Cell proliferation of EMSCs on GC scaffolds was detected by cell counting kit-8 (CCK8) assay. EMSCs (2000 cells/well) were cultured in 96-well plates. Cells were incubated for 24 h, 48 h, 72 h, and 96 h. Cells were then incubated with 10% CCK8 solution (Biyuntian, China) for 60 min. Absorbance was detected at 450 nm using an ELISA plate reader (Bio-Rad).

### 2.9. Scanning Electron Microscopy (SEM) Analysis

The morphologies of GC scaffolds were observed by SEM. In brief, the matrices were fixed with 2.5% glutaraldehyde solution and subsequently dehydrated in gradient ethanol. Before measurement, the samples were subjected to critical point vacuum drying and gold-spray treatment and were imaged by SEM (Quanta 200).

### 2.10. Immunofluorescence Staining

Cells or liver sections were fixed with 4% paraformaldehyde for 20 min, washed with PBS twice, and then permeabilized with 0.2% Triton X-100 (Sigma-Aldrich, USA) for 10 min at room temperature. After being blocked with 10% goat serum, cells were incubated in primary antibodies (1 : 200, Santa Cruz Biotechnology, USA) against *α*-fetoprotein (AFP), albumin (ALB), cytochrome P450 (CYP) 3A, and cytokeratin 18 (CK18) at 4°C overnight, followed by another 1 h of incubation in blocking solution with secondary antibodies at 37°C. Finally, the cells were stained with 4,6-diamidino-2-phenylindole (DAPI) (1 : 1000) for nuclear staining and visualized using a fluorescence microscope (Axio Vert A1).

### 2.11. Periodic Acid-Schiff (PAS) Staining for Glycogen

For glycogen storage detection of newly hepatocyte-like cells, PAS staining was performed. Briefly, samples were fixed with 4% paraformaldehyde for 30 min before being washed with PBS twice. These differentiated cells were then immersed in 1% periodic acid solution for 10 min and rinsed thrice in dH_2_O. Afterward, cell sides were treated with Schiff's reagent for 15 min, rinsed three times in dH_2_O, stained with hematoxylin for 1 min, and finally, rinsed in dH_2_O for 5 min, observed under an inverted microscope.

### 2.12. Evaluation of Urea Synthesis

To detect urea synthesis, cells (differentiated on days 7 and 14) were incubated with 5 mM/L NH_4_Cl for 24 h at 37°C according to a previous study [19]. The urea medium was collected and measured using a colorimetric assay (LABLEAD, China) according to the manufacturer's instructions.

### 2.13. Carbon Tetrachloride- (CCl_4_-) Induced Hepatocyte Injury In Vitro

The hepatotoxic injury was established according to the previous study [20]. Briefly, the rat IAR20 hepatocytes were incubated for 6 h with an addition of 8 mM CCl_4_ to induce liver injury. The cells were then incubated in fresh culture medium cocultured with EMSCs through a transwell chamber. The EMSC suspension was placed in the upper chamber, and the injured hepatocytes were placed in the lower chamber. The coculture supernatant samples were used to measure the levels of VEGF and HGF. Enzyme-linked immunosorbent assay (ELISA) was performed with commercial ELISA kits (Solaibao, China) according to the manufacturer's instructions.

### 2.14. Transplantation of EMSCs to CCL_4_-Induced ALF Rats

On day 0, the ALF rat (male, 220-280 g) model was established with a single intraperitoneal injection of CCL_4_ dissolved in olive oil (1 : 1) at the dose of 5 mL/kg body weight. On day 1, rats were transplanted with bare GC and EMSC-GC scaffolds, while the nontransplanted rats were set as a CCl_4_ group. The blank control group (control group) was not subjected to any treatment. There were 30 rats in each group. Pentobarbital was intraperitoneally injected into rats to deeply anesthetize them during the surgery. Incisions were made along the midline to expose the left lateral lobe of the liver. The fabric was sutured to the surface of the liver and secured with sutures.

### 2.15. Posttransplantation Liver Function Assessment

Alanine transaminase (ALT) and aspartate transaminase (AST) levels were determined in serum samples collected on the first, third, and seventh days after transplantation using ALT and AST assay kits (Nanjing Jiancheng Bioengineering Institute, China).

### 2.16. Pathological Examination

Hematoxylin and eosin (H&E) staining was performed using a Hematoxylin and Eosin Staining Kit (Solarbio, China). We collected liver tissue samples from different time points after transplantation and fixed them in 4% paraformaldehyde for 24 hours, followed by gradient dehydration. The samples were then embedded in paraffin. The tissues were cut into 5 *μ*m sections and stained with hematoxylin and eosin.

### 2.17. Histopathology and Immunohistochemistry

For immunohistochemical staining, after routine deparaffinization, rehydration, antigen retrieval, and endogenous peroxidase inactivation, samples were blocked with goat serum. Then, the sections were incubated with primary antibodies overnight at 4°C. Subsequently, the liver section slides were incubated with streptavidin-HRP, and this was followed by detection with DAB substrate. The antibodies were used at the following dilutions: HGF (1 : 100, Proteintech) and VEGF (1 : 100, Proteintech).

### 2.18. Statistical Analysis

SPSS 25.0 (SPSS Company, USA) and GraphPad Prism (GraphPad Prism 9.0, USA) were employed for statistical analysis. All numerical values presented in this study were mean ± standard error of the mean. Multiple group statistical analyses were performed by one-factor analysis of variance (ANOVA) tests. All statistically processed results were determined to be significant at ^∗^*p* < 0.05, ^∗∗^*p* < 0.01, and ^∗∗∗^*p* < 0.001.

## 3. Results

### 3.1. Phenotypic Characterization and Multiple Differentiation of EMSCs

**Figure 1:**
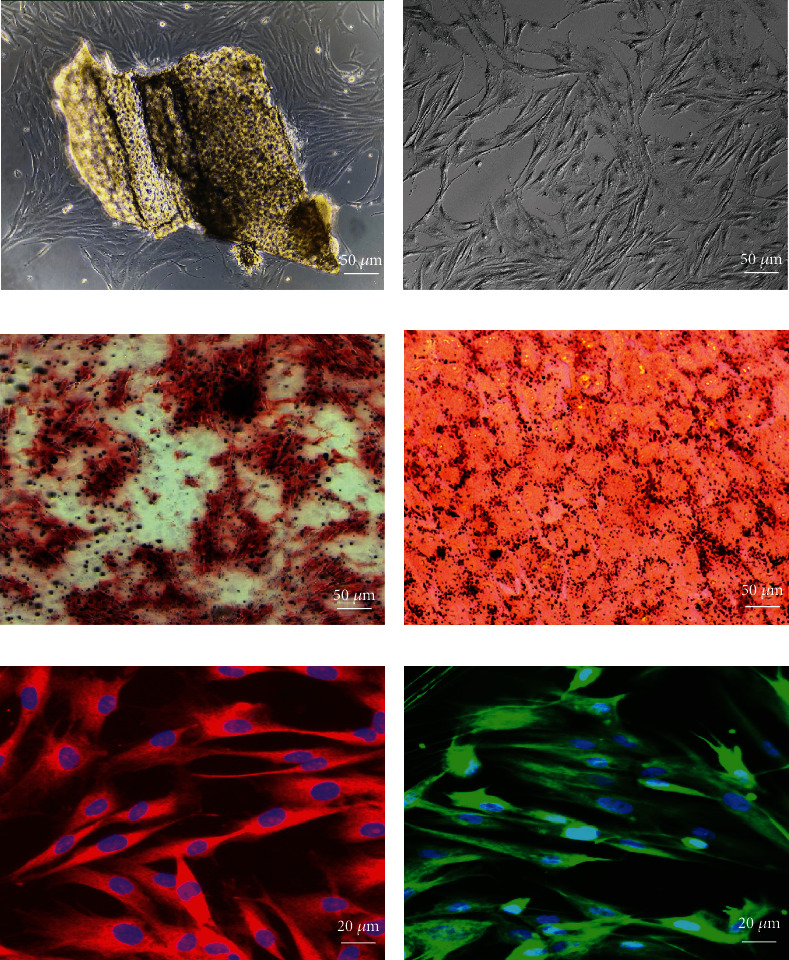
Isolation and identification of EMSCs. (a) P0 EMSCs. (b) P3 EMSCs. (c) Alizarin red staining shows calcium deposits and osteogenic differentiation. (d) Oil Red staining of intracellular accumulated lipids shows adipogenic differentiation. The EMSCs expressed the stem cell markers, including nestin (e) and vimentin (f).

On the second or third day of culture, the nasal mucosa-isolated cells formed spheroids and were suspended in the culture medium. The adherent spindle-shaped cells were observed on day 7. Partial cells crawled out of the nasal mucosa completed attachment against the wall of the flask and grew scattered (Figure 1(a)). The suspended cells declined in number as the media changed. For 15-20 days, the cells were centered around the tissue block and grew densely, until cells covered the bottom of the plastic flask. EMSCs were characterized in passage 3. The cells reached 80-90% confluence, and the morphology of passage 3 cells grew spindle-shaped and whirlpool with an oval nucleus (Figure 1(b)). The adipocytic and osteogenic differentiation of EMSCs was assessed by Oil Red O and alizarin red staining, respectively, to demonstrate the multipotent differentiation capability of target cells. Mineralization calcium nodus reaction showed positive with jacinth by alizarin red staining (Figure 1(c)). Oil Red O staining showed bright-red granular lipid droplets with various sizes of deposits in cells (Figure 1(d)). Immunofluorescence staining revealed that neural stem cell markers, including nestin (Figure 1(e)) and vimentin (Figure 1(f)), were positive.

### 3.2. Scaffold Architecture and Biocompatibility of EMSCs In Vitro

**Figure 2:**
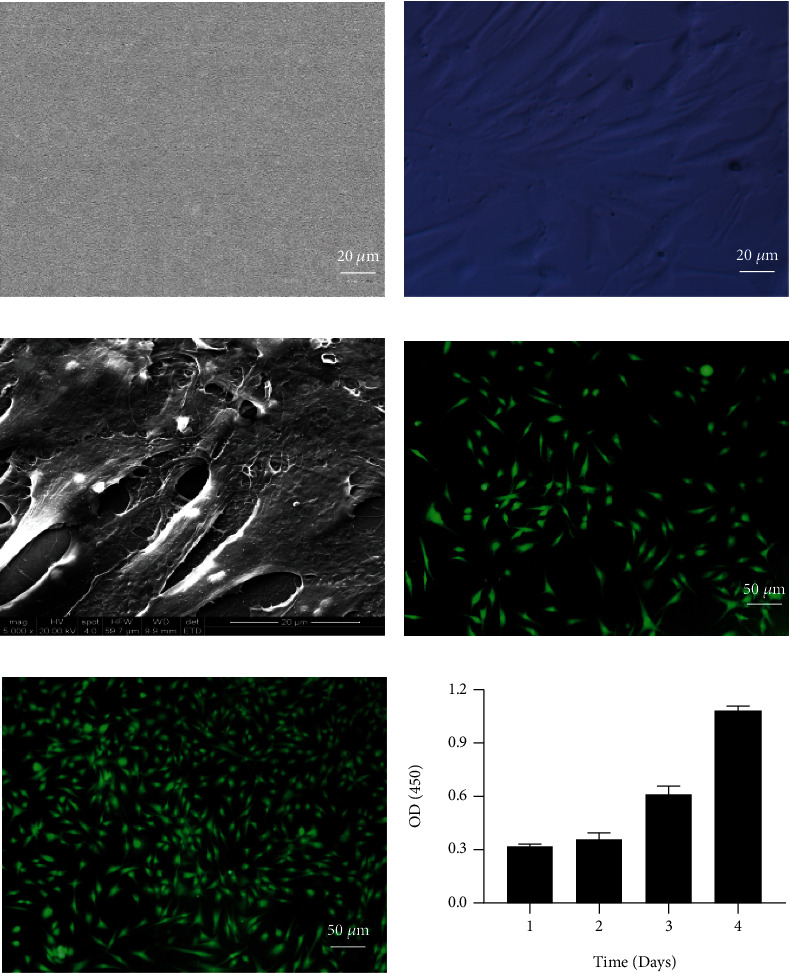
Morphological characteristics of EMSCs and the GC scaffold. (a) SEM images of the morphology of the GC scaffold. (b, c) Image of EMSCs in the GC scaffold as seen by optical microscopy (b) and SEM (c). (d, e) The viability of EMSCs in the GC scaffold was assessed using live/dead staining on day 2 (d) and day 5 (e) (green: live cells). (f) The cell proliferation ability was determined by the CCK8 method.

The EMSCs were seeded in 6-well dishes on GC scaffolds. SEM images revealed that the scaffolds had smooth, dense surfaces (Figure 2(a)). Scaffolds containing cells were transparent. The structure allows for easy visualization and observation of scaffolds and cells by light microscopy (Figure 2(b)) and SEM examination (Figure 2(c)). EMSCs were cultured, and cell viability was determined using live/dead assay kits on days 2 and 5. As shown in Figures 2(d) and 2(e), cells remained viable and increased largely during the culture period. EMSCs maintained an excellent adhesion and proliferation capacity on the surface of the GC scaffolds. We also measured the absorbance of cells on GC biofilm at different time points through proliferation assay using CCK8. EMSCs demonstrated enhanced cell proliferation over time (Figure 2(f)). Taken together, these results indicated good biocompatibility between EMSCs and GC scaffolds.

### 3.3. Improvement of EMSCs in CCl_4_-Induced Acute Liver Failure

**Figure 3:**
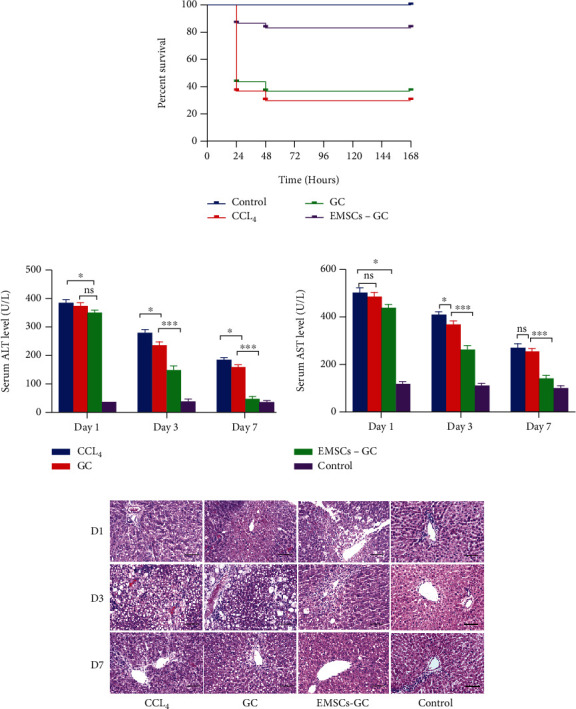
EMSC transplantation increased survival and reduced liver injury in ALF rats. (a) Survival analysis of each group. (b) Liver function analysis in CCl_4_-treated rats on days 1, 3, and 7 after GC and EMSC-GC matrices. (c) Representative liver tissue pathology H&E staining (200x magnification) on different days after GC and EMSC-GC matrix transplantation. The EMSC-GC matrix groups show smaller congestion areas than the CCl_4_ group. Scale bar = 200 *μ*m.

To confirm the therapeutic potential of EMSC-GC on liver regeneration and repair, SD rats were administered intraperitoneally with CCl_4_ to generate acute fulminant hepatic injury. All CCl_4_-stimulated animals developed severe liver enlargement and ascites 24 hours later. GC scaffolds or EMSC-GC was transplanted to the surface of the livers of rats with ALF. Serum ALT and AST levels were measured at various time points to assess liver function. In survival analysis, none of the animals in the control group died. 25/30 animals treated with EMSC-GC survived, compared to fewer rats (11/30) in the GC group and only 9/30 rats in the CCl_4_ group (Figure 3(a)). These results demonstrated that EMSCs significantly increased the survival rate of ALF rats. Compared to those in the control group, the levels of ALT and AST were significantly higher in CCl_4_-treated rats. However, serum levels of ALT and AST significantly declined 3 days after the transplantation of EMSC-GC (Figure 3(b)). In addition, the GC group displayed a certain therapeutic effect, but much weaker than that of the EMSC-GC matrix groups. On day 7, the functions of injured rats receiving EMSC-GC transplantation showed nearly normal levels of liver function. Remarkably, H&E staining also confirmed the therapeutic potential of EMSC-GC in reducing liver damage (Figure 3(c)). After 3 days of CCl_4_ injection, the CCl_4_-treated rats displayed severe hepatocyte necrosis, extensive fatty vacuolar degeneration, and inflammatory cell infiltration compared to the control group. However, the congestion areas of the EMSC-GC scaffold group were smaller than those of the CCl_4_ group and GC group. On day 7, the EMSC-GC group was almost histologically recovered. Taken together, our results showed that transplantation of EMSCs via GC scaffolds significantly increased survival rates, ameliorated liver transaminases, and histological abnormalities.

### 3.4. Biocompatibility of EMSC-GC on Liver Surface In Vivo

**Figure 4:**
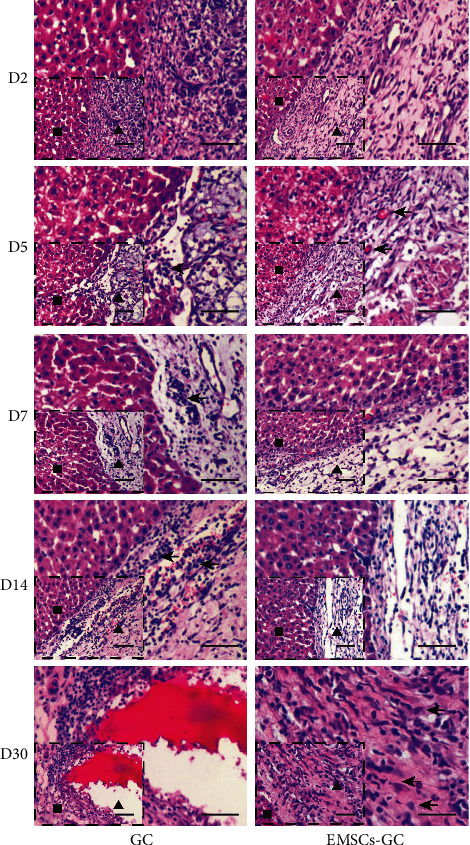
H&E staining of GC and EMSC-GC scaffolds on the liver in vivo. Small insets depict an overview of the GC scaffolds and liver tissues. The border between the GC scaffolds and the liver parenchyma is represented by larger figures. Day 2: the borders of the GC matrices (the triangle) and the liver parenchyma (the square) were clear in the sections. Day 5: neovascularization was observed in the EMSC-GC groups (arrowhead). Day 7: the number of new blood vessels decreased, whereas the inflammatory cells markedly infiltrated the control group (arrowhead). Day 14: the neovascularized areas receded with limited inflammatory cell infiltration in the EMSC-GC group and a large amount of inflammatory cell infiltration in the control group. Month 1: in the EMSC-GC groups, some hepatocyte-like cells with large nuclei, abundant cytoplasm, and light color were observed at the edges of the scaffold (arrowhead). Scale bar = 200 *μ*m.

To test the applicability of implanted tissue scaffolds in vivo, GC and EMSC-GC were implanted into the liver of an acute liver failure model. Different time points were observed for the compatibility of GC materials with the livers, as well as the differentiation of EMSCs transplanted into the liver (Figure 4). On day 2, H&E staining revealed that EMSC-GC scaffolds adhered to the liver surface, with EMSCs positioned over GC scaffolds. In the bare material treated group, small amounts of inflammatory cells started to infiltrate. On day 5, neovascularization was evident in the EMSC-GC group. Compared to day 2, the overall number of inflammatory cells increased. On day 7, angiogenesis began to decrease and the borders between the transplanted scaffold and the liver parenchyma were still clear. There was more remarkable inflammatory cell infiltration in the blank GC scaffold group. On day 14, the neovascularization disappeared, accompanied by a small infiltration of inflammatory cells in the EMSC-GC groups. However, the control group still revealed abundant inflammatory cell infiltration. In the EMSC-GC groups, the cell proliferated clearly at the junctional zone one month following implantation. Notably, there were several hepatocyte-like cells with a large nucleus, copious cytoplasm, and light color at the scaffold's boundaries. The GC scaffold in the control group degraded, and the junction between materials and liver parenchyma was evident; however, inflammatory cells were clearly visible. CCl_4_-induced liver injury provides a damaged environment for the hepatocytes which may advance hepatic differentiation of EMSCs in vivo.

### 3.5. EMSCs Showed High Levels of VEGF and HGF Release in CCL_4_-Induced Hepatocyte Injury

**Figure 5:**
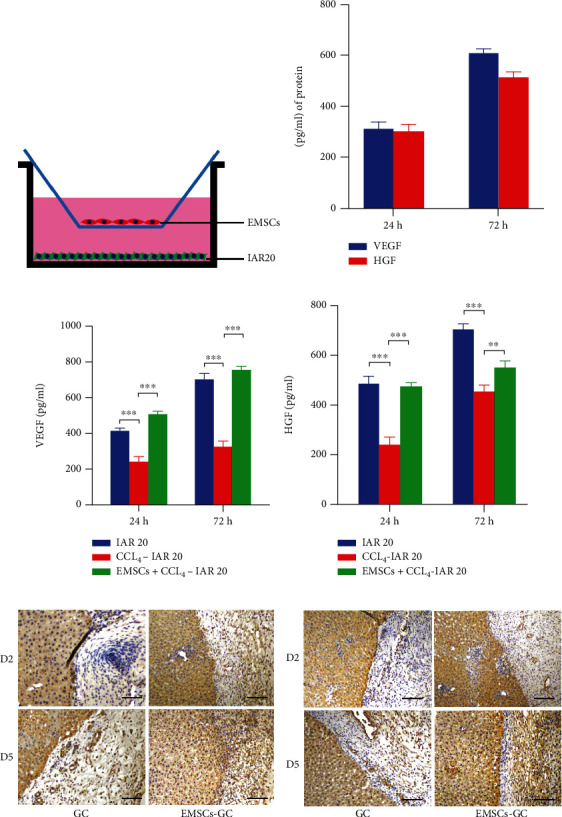
EMSCs protected CCl_4_-induced hepatocyte injury by secreting VEGF and HGF in vitro and in vivo. (a) Schematic illustration of coculture assay. (b) Secretion of VEGF and HGF in the culture media of EMSCs. (c, d) EMSC coculture significantly increased VEGF and HGF levels compared to the CCl_4_ treatment group. (e, f) Immunohistochemical examination of VEGF (e) and HGF (f) expressions in vivo. Scale bar = 200 *μ*m.

Some protective factors such as VEGF [21, 22] and HGF [23, 24] have been reported to enhance hepatocyte regeneration and repair liver injury. To explore the potential mechanism of stem cells protecting damaged hepatocytes, we evaluated the paracrine effect of EMSCs through the coculture assay. EMSCs showed the increased secretory effect of VEGF and HGF with time when cultured alone (Figure 5(b)). Following CCl_4_ administration, the levels of VEGF and HGF in the culture supernatants were elevated significantly in the EMSC groups, whereas the release of these proteins was suppressed in the injury group (Figures 5(c) and 5(d)). Likewise, a high concentration of VEGF and HGF in the EMSC-GC groups was noticed as compared to the injury GC group detected by the liver sections. As determined by immunohistochemistry, EMSCs released a tiny quantity of VEGF and HGF on day 2, while by day 5, they had nearly reached the same level of secretion as liver parenchyma (Figures 5(e) and 5(f)). This may help to explain the reason of EMSCs repairing hepatocyte injury to a certain extent.

### 3.6. EMSCs Could Autodifferentiate into HLCs In Vivo and In Vitro

**Figure 6:**
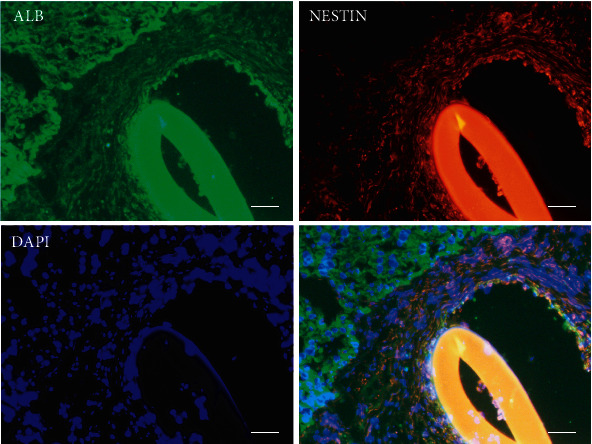
Transplanted EMSC-GC engraft in the rat and can differentiate into HLCs. Hepatic differentiation in vivo was confirmed through immunofluorescent staining with the stem cell marker (nestin) and the hepatic differentiation marker (ALB) and examined under a microscope. Scale bar = 25 *μ*m.

Many studies have already shown that most of the transplanted MSCs will undergo apoptosis within a week [25], and a few surviving MSCs will home in the liver tissue and transdifferentiate into hepatocytes [26]. As a potential source of cell therapy for liver diseases, the fate of transplanted EMSCs in vivo is a significant issue. One month after EMSC transplantation, we observed a layer of dense cell clusters on the surface of rat liver, which featured as HLCs with large nuclei, abundant cytoplasm, and light color. In addition, hepatic differentiation in vivo was confirmed through the detection of the stem cell marker (nestin) and the hepatic differentiation marker (ALB) by immunofluorescence staining (Figure 6). EMSCs slowly shed from the scaffolds and migrated to the liver parenchyma and proliferated in the area between the scaffolds and liver parenchyma. The local microenvironment of liver injury promoted the differentiation of EMSCs into hepatocyte-like cells [27].

**Figure 7:**
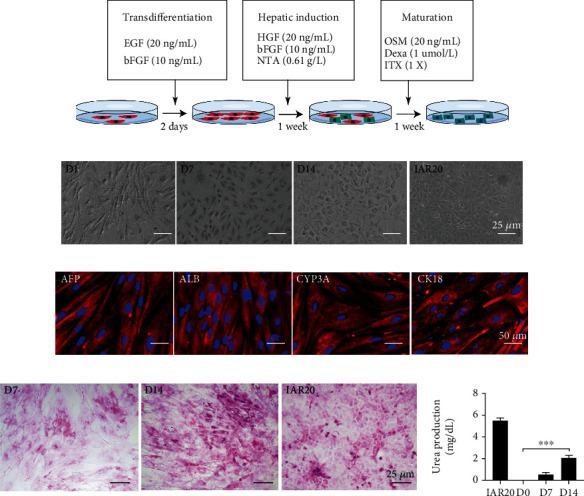
Induction EMSCs into HLCs in vitro. (a) Schematic diagram of EMCSs' differentiation into HLCs. (b) Morphological changes of undifferentiated EMSCs to differentiated hepatocyte-like cells under hepatogenic conditions. (c) Fluorescent images of hepatic markers of AFP, ALB, CYP3A, and CK18. (d) PAS staining and urea production of differentiated hepatocyte-like cells.

Moreover, to test whether EMSCs could be induced into HLCs in vitro, cells were exposed to the hepatogenic differentiation medium by a three-step, 14-day protocol as described in Figure 7(a). Morphological changes of EMSCs during differentiation were observed by phase-contrast microscopy. Two weeks after treatment, the cultivated cells all responded effectively to treatment and converted into small round or polygonal shapes (Figure 7(b)), similar to rat hepatocytes. We define these cells as hepatocyte-like cells (HLCs). Cell immunofluorescence analysis showed that AFP was expressed in the early stage of differentiation. On day 14, the mature hepatocyte markers, ALB, CYP3A, and CK18, were detected in hepatocyte-like cells (Figure 7(c)). In addition, we measured glycogen synthesis and urea production to test whether the HLCs possess a function similar to that of mature hepatocytes. The red granules by PAS staining indicated the glycogen storage capacity of HLCs. Ammonia-induced urea secretion is another important function of mature hepatocytes. Urea assays showed that mature hepatocytes were capable of removing ammonia from the culture medium when stimulated with NH_4_Cl. The urea concentration gradually increased during the differentiation period from a value of 0.52 ± 0.06 mg/dL on day 7 of differentiation to 2.05 ± 0.52 mg/dL on day 14 of differentiation (Figure 7(d)). These findings demonstrated that we were able to acquire EMSC-derived hepatocytes in 14 days utilizing a nontransgenic technique. EMSC-derived HLCs functioned similarly to matured primary hepatocytes.

## 4. Discussion

ALF, a fatal clinical disorder with severe hepatic damage, is associated with a high mortality rate [1]. MSC therapy is an emerging promising therapeutic approach for liver failure. In recent years, considerable preclinical and clinical research has proven that MSCs including bone marrow and umbilical cord-derived stem cells can effectively relieve liver injury and regenerate hepatocytes [28–31]. Current problems in the application of clinical treatment of MSCs are limited sources, low activity, and less colonization in target organs [32]. EMSCs are multipotent stem cells derived from the cranial neural crest [33]. They can be obtained easily and minimally invasively from the olfactory mucosa [34]. In our study, EMSCs exhibited a multiple-lineage differentiation ability into adipocytes and osteocytes. Those cells highly expressed neural crest cell and mesenchymal stem cell markers, including nestin and vimentin. The above characteristics make EMSCs a potential source of stem cells for cell-based therapeutic strategies. To our knowledge, no attempts have so far been made to determine whether EMSCs can transdifferentiate into HLCs, improve liver function, and rescue the ALF. In this study, we reported that EMSCs have the potential to differentiate into mature hepatocytes in vitro using a three-step protocol. This process took just 14 days to generate cells that show hepatocyte-specific morphology and functionality. More importantly, transplantation of EMSCs significantly ameliorated carbon tetrachloride-induced liver injury models. The transplanted cells developed into hepatocytes in vivo without additional growth factors or cytokines.

Decreased localized cells, circulatory embolism, and immunological rejection are the primary negative effects of stem cell transplantation [35]. To address these issues, EMSCs were seeded into GC scaffolds and then transplanted onto the liver surface of ALF rats. The scaffolds provide an excellent environment for the adherence and proliferation of MSCs [36]. We found that EMSCs attached tightly and proliferated quickly on the scaffolds, confirming previous studies indicating that chitosan was a very biocompatible material for cell attachment and growth [36]. Liver function and histopathology were significantly improved in the EMSC-GC transplantation group as compared to the CCl_4_ or GC group. H&E staining of liver sections from EMSC-transplanted ALF rats revealed fewer areas of necrosis and inflammatory cell infiltration, and hepatocyte regeneration was apparent. Most importantly, transplanted EMSC treatment significantly reduced the mortality in CCl_4_-irradiated acute liver failure. The follow-up results revealed that a high amount of EMSCs proliferated on the liver surface. The number of inflammatory cells was reduced, and more neovascularization was observed. These results indicated a great therapeutic value of EMSCs in treating ALF.

Previous studies showed that the main mechanisms of the transplantation of MSCs repairing liver injury are involved mainly in the paracrine effect and hepatogenic differentiation. Liu et al. found that MSCs decreased hepatocyte apoptosis and promoted liver regeneration through secretory cytokines and growth factors [37]. Zhou et al. found that HLCs derived from the induction of MSCs have a significant benefit and targeted migration to liver injury sites [38]. Therefore, we quantified the levels of VEGF and HGF in the supernatant of EMSC and damaged hepatocyte coculture medium. The results confirmed that VEGF and HGF levels in the EMSC-cocultured group were significantly higher than in the CCl_4_ exposure group. VEGF stimulated angiogenesis and wound healing as a growth factor. When injected into partially hepatectomy rat livers, exogenous VEGF promotes the proliferation of hepatocytes, showing that VEGF is important in liver regeneration [39]. HGF is the most potent liver mitogen that initiates hepatic regeneration and acts directly on hepatocytes [40]. In vivo experiments further proved the protective effect of EMSCs by secretory cytokines. EMSCs detached from the substrates and migrated to the gap between the liver parenchyma and the scaffolds, distributing along the surface of the liver parenchyma. The injured liver microenvironment promoted the chemotaxis and orientation of stem cells from scaffolds to liver parenchyma.

Intriguingly, HLCs with large nuclei, abundant cytoplasm, and light color were found in the repair period of injured rats. Xu et al. also reported a similar outcome, finding that bone marrow or adipose-derived MSCs loaded with a silk fibroin scaffold could differentiate into HLCs in rat models of acute liver failure [15]. Thus, we detected the hepatogenic differentiation of EMSCs in vivo by immunofluorescence, and EMSCs highly expressed the hepatocyte marker ALB. This demonstrated that EMSCs are capable of transdifferentiating into HLCs and their short-term application in vivo is feasible. Regulatory mechanisms that induce MSCs to differentiate into hepatic lineage may have tight ties to the specific niche environment. A previous study has revealed that MSCs cocultured with liver cells from injured rats could form round or polygonal HLCs and expressed liver markers on both the molecular and protein levels [41]. Meanwhile, transplanted MSCs differentiated into hepatocytes in vivo by injection into the spleen or the tail vein of mice with liver injury [42, 43]. Furthermore, to test whether MSCs could be successfully inducted into HLCs, in vitro hepatic differentiation was performed. The main hepatic differentiation protocols of MSCs can be summarized as follows: addition of cytokines and growth factors, genetic modifications, and adjustment of microenvironment and physical parameters [44–47]. In the present study, we utilized the most commonly used method: combined treatment with different cytokines and growth factors in separate steps. During the primary induction step, EMSCs were treated with EGF and bFGF to transdifferentiate into endodermal cells. EGF is a growth factor that may mediate the proliferation and differentiation of MSCs by binding its receptor EGFR [48]. Similar to EGF, bFGF displays a promotion effect in the proliferation course [48]. To further facilitate hepatogenic maturation, HGF, NTA, ITS, OSM, and Dexa were added to the culture medium. HGF is a pleiotropic growth factor that functions in liver biology and the hepatogenesis process. It is also involved in regulating cell proliferation and further differentiation of MSCs by interacting with cMet receptor and activating the downstream effectors ERK1/2, p38MAPK, and PI3K/Akt [49]. Furthermore, NTA and ITS may have a similar potency to enhance the proliferation of MSCs and survival maintenance of primary hepatocytes [50]. The addition of OSM further develops hepatic cells to maturation steps. It might be directly correlated with the downregulation of Sox9, which is a critical gene for hepatocyte lineage development [51]. Dexamethasone increases the enzyme activity of glycogen synthesis in the liver and strongly enhances albumin expression and cytochrome P450 levels [52]. In this paper, we established the three-step method for the differentiation of EMSCs into hepatocytes. When EMSCs were cultured in proliferation cytokines (EGF and bFGF) in combination with maturation factors (HGF, OSM, Dexa, and ITS), cells showed morphology and functions of mature hepatocytes. Based on our results, EMSCs expressed a high level of AFP on day 7. This is an evaluation indicator for hepatogenic differentiation in the early stage because AFP is specifically expressed in the hepatocyte progenitor population [53]. Moreover, differentiated EMSCs retained the properties of mature hepatocytes, including albumin production, glycogen storage, and drug-metabolizing enzyme CYP3A activity.

So, we speculate that there are two possible reasons for the protective effect of the transplantation of EMSCs. (1) Transplanted EMSCs may secrete paracrine factors to promote hepatocyte regeneration and targeted migration in the short term. (2) Transplanted EMSCs may transdifferentiate into HLCs in response to the liver's microenvironment, which will then repair or replace the damaged hepatocyte in the long term.

## 5. Conclusions

Taken together, EMSCs exhibit the characteristics of easy availability, minimal damage, and autologous transplantation. We have identified that EMSCs could be induced to generate functional HLCs in vitro and in vivo. GC scaffold-loaded EMSC treatment remarkably increases survival, improves liver function, and alleviates pathological histology in the acute liver failure model by secreting protective factors. These results strongly suggest that EMSCs have great potential for use as novel seed cells in treating liver failure.

## Data Availability

All data generated or analyzed during this study are included in this published article.

## References

[1] Stravitz R. T., Lee W. M. (2019). Acute liver failure. *Lancet*.

[2] Volarevic V., Nurkovic J., Arsenijevic N., Stojkovic M. (2014). Concise review: therapeutic potential of mesenchymal stem cells for the treatment of acute liver failure and cirrhosis. *Stem Cells*.

[3] Tao Y. C., Wang M. L., Chen E. Q., Tang H. (2018). Stem cells transplantation in the treatment of patients with liver failure. *Current Stem Cell Research & Therapy*.

[4] Liu W. H., Song F. Q., Ren L. N. (2015). The multiple functional roles of mesenchymal stem cells in participating in treating liver diseases. *Journal of Cellular and Molecular Medicine*.

[5] Hu C., Li L. (2019). Improvement of mesenchymal stromal cells and their derivatives for treating acute liver failure. *Journal of Molecular Medicine (Berlin, Germany)*.

[6] Huang B., Cheng X., Wang H. (2016). Mesenchymal stem cells and their secreted molecules predominantly ameliorate fulminant hepatic failure and chronic liver fibrosis in mice respectively. *Journal of Translational Medicine*.

[7] Gilsanz C., Aller M. A., Fuentes-Julian S. (2017). Adipose-derived mesenchymal stem cells slow disease progression of acute-on- chronic liver failure. *Biomedicine & Pharmacotherapy*.

[8] Tautenhahn H. M., Bruckner S., Baumann S. (2016). Attenuation of postoperative acute liver failure by mesenchymal stem cell treatment due to metabolic implications. *Annals of Surgery*.

[9] Paino F., Ricci G., De Rosa A. (2010). Ecto-mesenchymal stem cells from dental pulp are committed to differentiate into active melanocytes. *European Cells & Materials*.

[10] Delorme B., Nivet E., Gaillard J. (2010). The human nose harbors a niche of olfactory ectomesenchymal stem cells displaying neurogenic and osteogenic properties. *Stem Cells and Development*.

[11] Zhang Z., Li Z., Deng W. (2016). Ectoderm mesenchymal stem cells promote differentiation and maturation of oligodendrocyte precursor cells. *Biochemical and Biophysical Research Communications*.

[12] Shi W., Que Y., Zhang X. (2021). Functional tissue-engineered bone-like graft made of a fibrin scaffold and TG2 gene-modified EMSCs for bone defect repair. *NPG Asia Materials*.

[13] Christ B., Bruckner S., Winkler S. (2015). The therapeutic promise of mesenchymal stem cells for liver restoration. *Trends in Molecular Medicine*.

[14] Si Y. L., Zhao Y. L., Hao H. J., Fu X. B., Han W. D. (2011). MSCs: biological characteristics, clinical applications and their outstanding concerns. *Ageing Research Reviews*.

[15] Xu L., Wang S., Sui X. (2017). Mesenchymal stem cell-seeded regenerated silk fibroin complex matrices for liver regeneration in an animal model of acute liver failure. *ACS Applied Materials & Interfaces*.

[16] Yang M. C., Wang S. S., Chou N. K. (2009). The cardiomyogenic differentiation of rat mesenchymal stem cells on silk fibroin-polysaccharide cardiac patches *in vitro*. *Biomaterials*.

[17] Shi W., Bian L., Lv D. (2021). Enhanced neural differentiation of neural stem cells by sustained release of Shh from TG2 gene-modified EMSC co-culture in vitro. *Amino Acids*.

[18] Zheng G., Liu Y., Jing Q., Zhang L. (2015). Differentiation of human umbilical cord-derived mesenchymal stem cells into hepatocytes in vitro. *Bio-medical Materials and Engineering*.

[19] Zhang S., Chen L., Liu T. (2012). Human umbilical cord matrix stem cells efficiently rescue acute liver failure through paracrine effects rather than hepatic differentiation. *Tissue Engineering. Part A*.

[20] Wu Y. H., Zhang X. M., Hu M. H., Wu X. M., Zhao Y. (2009). Effect of Laggera alata on hepatocyte damage induced by carbon tetrachloride in vitro and in vivo. *Journal of Ethnopharmacology*.

[21] Ma T., Liu H., Chen W. (2012). Implanted adipose-derived stem cells attenuate small-for-size liver graft injury by secretion of VEGF in rats. *American Journal of Transplantation*.

[22] Adas G., Koc B., Adas M. (2016). Effects of mesenchymal stem cells and VEGF on liver regeneration following major resection. *Langenbeck's Archives of Surgery*.

[23] Shams S., Mohsin S., Nasir G. A., Khan M., Khan S. N. (2015). Mesenchymal stem cells pretreated with HGF and FGF4 can reduce liver fibrosis in mice. *Stem Cells International*.

[24] Kim M. D., Kim S. S., Cha H. Y. (2014). Therapeutic effect of hepatocyte growth factor-secreting mesenchymal stem cells in a rat model of liver fibrosis. *Experimental & Molecular Medicine*.

[25] Eggenhofer E., Benseler V., Kroemer A. (2012). Mesenchymal stem cells are short-lived and do not migrate beyond the lungs after intravenous infusion. *Frontiers in Immunology*.

[26] Zhang G. Z., Sun H. C., Zheng L. B., Guo J. B., Zhang X. L. (2017). In vivo hepatic differentiation potential of human umbilical cord-derived mesenchymal stem cells: therapeutic effect on liver fibrosis/cirrhosis. *World Journal of Gastroenterology*.

[27] Sun H., Shi C., Ye Z. (2022). The role of mesenchymal stem cells in liver injury. *Cell Biology International*.

[28] Li Y. H., Xu Y., Wu H. M., Yang J., Yang L. H., Yue-Meng W. (2016). Umbilical cord-derived mesenchymal stem cell transplantation in hepatitis B virus related acute-on-chronic liver failure treated with plasma exchange and entecavir: a 24-month prospective study. *Stem Cell Reviews and Reports*.

[29] Lin B. L., Chen J. F., Qiu W. H. (2017). Allogeneic bone marrow–derived mesenchymal stromal cells for hepatitis B virus–related acute-on-chronic liver failure: a randomized controlled trial. *Hepatology*.

[30] Shi M., Zhang Z., Xu R. (2012). Human mesenchymal stem cell transfusion is safe and improves liver function in acute-on-chronic liver failure patients. *Stem Cells Translational Medicine*.

[31] Peng L., Xie D. Y., Lin B. L. (2011). Autologous bone marrow mesenchymal stem cell transplantation in liver failure patients caused by hepatitis B: short-term and long-term outcomes. *Hepatology*.

[32] Lee C. A., Sinha S., Fitzpatrick E., Dhawan A. (2018). Hepatocyte transplantation and advancements in alternative cell sources for liver-based regenerative medicine. *Journal of Molecular Medicine (Berlin, Germany)*.

[33] Zhang Z., He Q., Deng W. (2015). Nasal ectomesenchymal stem cells: multi-lineage differentiation and transformation effects on fibrin gels. *Biomaterials*.

[34] Ibarretxe G., Crende O., Aurrekoetxea M., Garcia-Murga V., Etxaniz J., Unda F. (2012). Neural crest stem cells from dental tissues: a new hope for dental and neural regeneration. *Stem Cells International*.

[35] Wang X., Li Z., Cui Y., Cui X., Chen C., Wang Z. (2021). Exosomes isolated from bone marrow mesenchymal stem cells exert a protective effect on osteoarthritis via lncRNA LYRM4-AS1-GRPR-miR-6515-5p. *Frontiers in Cell and Development Biology*.

[36] He J., Wu F., Wang D., Yao R., Wu Y., Wu F. (2015). Modulation of cationicity of chitosan for tuning mesenchymal stem cell adhesion, proliferation, and differentiation. *Biointerphases*.

[37] Liu Z., Meng F., Li C. (2014). Human umbilical cord mesenchymal stromal cells rescue mice from acetaminophen- induced acute liver failure. *Cytotherapy*.

[38] Zhou R., Li Z., He C. (2014). Human umbilical cord mesenchymal stem cells and derived hepatocyte-like cells exhibit similar therapeutic effects on an acute liver failure mouse model. *PLoS One*.

[39] Gnecchi M., Zhang Z., Ni A., Dzau V. J. (2008). Paracrine mechanisms in adult stem cell signaling and therapy. *Circulation Research*.

[40] Bansal M. B. (2016). Hepatic stellate cells: fibrogenic, regenerative or both? Heterogeneity and context are key. *Hepatology International*.

[41] Qihao Z., Xigu C., Guanghui C., Weiwei Z. (2007). Spheroid formation and differentiation into hepatocyte-like cells of rat mesenchymal stem cell induced by co-culture with liver cells. *DNA and Cell Biology*.

[42] Li T. Z., Kim J. H., Cho H. H. (2010). Therapeutic potential of bone-marrow-derived mesenchymal stem cells differentiated with growth-factor-free coculture method in liver-injured rats. *Tissue Engineering. Part A*.

[43] Yu J., Cao H., Yang J. (2012). In vivo hepatic differentiation of mesenchymal stem cells from human umbilical cord blood after transplantation into mice with liver injury. *Biochemical and Biophysical Research Communications*.

[44] Stock P., Bruckner S., Ebensing S., Hempel M., Dollinger M. M., Christ B. (2010). The generation of hepatocytes from mesenchymal stem cells and engraftment into murine liver. *Nature Protocols*.

[45] Lange C., Bassler P., Lioznov M. V. (2005). Hepatocytic gene expression in cultured rat mesenchymal stem cells. *Transplantation Proceedings*.

[46] Talens-Visconti R., Bonora A., Jover R. (2006). Hepatogenic differentiation of human mesenchymal stem cells from adipose tissue in comparison with bone marrow mesenchymal stem cells. *World Journal of Gastroenterology*.

[47] Afshari A., Shamdani S., Uzan G., Naserian S., Azarpira N. (2020). Different approaches for transformation of mesenchymal stem cells into hepatocyte-like cells. *Stem Cell Research & Therapy*.

[48] Salehinejad P., Alitheen N. B., Mandegary A., Nematollahi-Mahani S. N., Janzamin E. (2013). Effect of EGF and FGF on the expansion properties of human umbilical cord mesenchymal cells. *In Vitro Cellular & Developmental Biology. Animal*.

[49] Forte G., Minieri M., Cossa P. (2006). Hepatocyte growth factor effects on mesenchymal stem cells: proliferation, migration, and differentiation. *Stem Cells*.

[50] Hong S. H., Gang E. J., Jeong J. A. (2005). In vitro differentiation of human umbilical cord blood-derived mesenchymal stem cells into hepatocyte-like cells. *Biochemical and Biophysical Research Communications*.

[51] Paganelli M., Nyabi O., Sid B. (2014). Downregulation of Sox9 expression associates with hepatogenic differentiation of human liver mesenchymal stem/progenitor cells. *Stem Cells and Development*.

[52] Ma X., Duan Y., Jung C. J., Wu J., VandeVoort C. A., Zern M. A. (2008). The differentiation of hepatocyte-like cells from monkey embryonic stem cells. *Cloning and Stem Cells*.

[53] Zhao R., Duncan S. A. (2005). Embryonic development of the liver. *Hepatology*.

